# Pancytopenia as an early indicator for Stevens-Johnson syndrome complicated with hemophagocytic lymphohistiocytosis: a case report

**DOI:** 10.1186/1471-2431-14-38

**Published:** 2014-02-10

**Authors:** Zhi-Dan Fan, Xiao-Qing Qian, Hai-Guo Yu

**Affiliations:** 1 Department of Rheumatology and Immunology, Nanjing Children’s Hospital Affiliated to Nanjing Medical University, No. 72 Guangzhou Road, Nanjing, Jiangsu Province 210008, China

**Keywords:** Pancytopenia, Early diagnosis, Stevens-Johnson syndrome (SJS), Hemophagocytic lymphohistiocytosis (HLH)

## Abstract

**Background:**

Stevens-Johnson syndrome (SJS) is a severe skin and mucosal bullous disease. When complicated with Hemophagocytic lymphohistiocytosis (HLH), the condition is especially life-threatening.

**Case presentation:**

Here we report the case of a 4-year-old boy suffering from SJS with extensive erythema multiforme and bulla. Despite active intervention and supportive care, the boy experienced increased skin lesions and a higher fever. Meanwhile, decreases in white blood cell count and hemoglobin were observed. Hyperferritinemia, increased soluble CD25 level, decreased NK cell activity and hemophagocytosis in the boy’s bone marrow confirmed the diagnosis of HLH. After high-dose intravenous immunoglobulin and methylprednisone pulse therapy, the boy was discharged in good condition.

**Conclusion:**

Simultaneous occurrence of HLH and SJS is very uncommon and the condition is life-threatening. Pancytopenia can be a precocious indicator and enables to start a prompt diagnosis and treatment.

## Background

Stevens-Johnson syndrome (SJS) is characterized morphologically by the rapid onset of epidermal detachment and erosion of the mucous membrane. Epithelial loss and the subsequent bacterial and fungal infections are the most common cause of death in patients with SJS [1]. Immune dysregulation plays a key role in the pathogenesis of SJS. The death-inducing interaction of Fas with its Fas ligand triggers a suicidal caspase cascade in epidermal cells. High-dose intravenous immunoglobulin (IVIG) was shown to contain natural Fas-blocking antibodies and thus abrogate the keratinocyte apoptosis and the subsequent epidermal detachment [2].

Hemophagocytic lymphohistiocytosis (HLH), a lethal immune disorder, often leads to an abrupt onset of single organ failure and rapid progression to multiple organ failure [3]. Unlike familial HLH due to genetic defects leading to impaired functions of natural killer and cytotoxic T cells, secondary HLH is generally triggered by infections, immunosuppressant drugs, malignant or rheumatologic disorders [4]. So far, in patients with SJS, HLH has been rarely reported [5]. However, SJS complicated with HLH is a life-threatening condition as the diagnosis often occurs too late to start timely life-saving therapy. Here we report a 4-year-old boy who developed HLH following SJS. The laboratory tests of pancytopenia can be a precocious indicator for us to make a prompt diagnosis, and to start a timely treatment to ensure the successful outcome.

## Case presentation

A previously healthy 4-year-old boy was admitted to our pediatric intensive care unit with a one-week history of spiking fever, coughing and a three-day history of severe mucous membrane and skin lesions. He received some cephalosporin and ibuprofen in another hospital for acute upper respiratory tract infection. However, after four days of treatment, he developed a higher fever and a raised erythematous rash.

**Figure 1:**
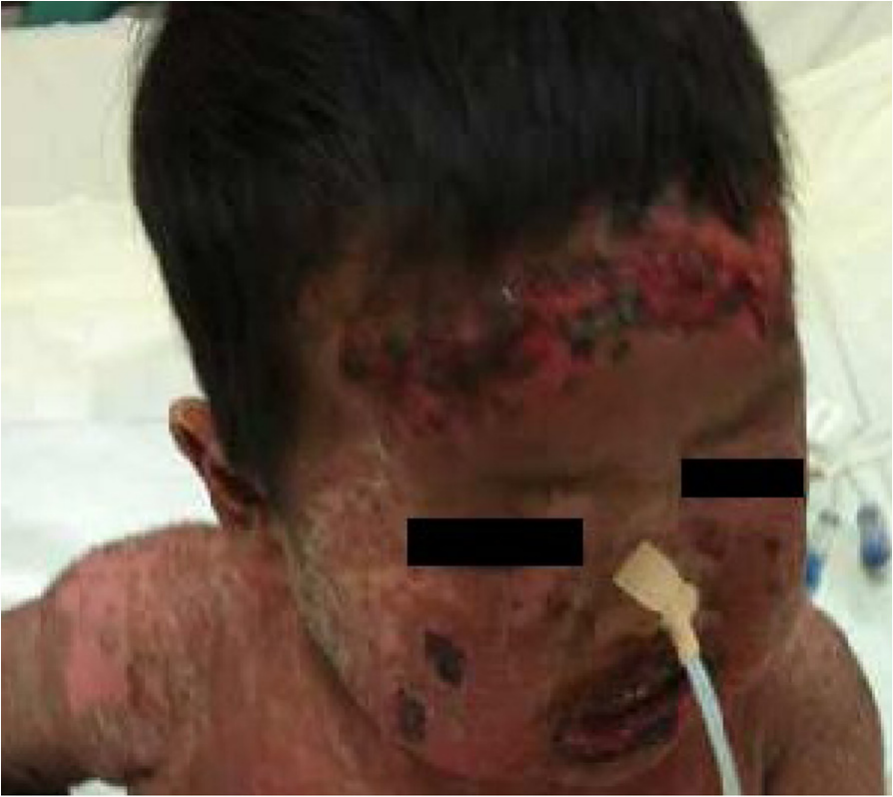
Serious oral mucosal erosions, vesicobullous lesions and skin detachment across the body.

On admission, the boy appeared unwell with temperature above 39°C and heart rate of 192 beats/min. The examination showed oral mucositis, vesicobullous lesions and skin detachment across the body (Figure 1).

Routine blood tests were unremarkable on admission. Biochemistry parameters were normal except for albumin (27 g/L [normal 35–55]) and serum sodium (126 mmol/L [normal 135–145]). Both erythrocyte sedimentation rate (61 mm/h [normal < 20]) and C-reactive protein (85 mg/L [normal < 10]) were markedly increased. The pathological and bacteriologic examinations indicated the absence of viral, mycoplasma pneumoniae and chlamydia pneumoniae infections and negative results of blood culture and galactomannan test.

The active intervention, anti-infection measures, fluid compensation, electrolyte balance, nutritional support and intensive care were initiated immediately after hospitalization with SJS. Meanwhile, high-dose IVIG (2 g/kg, single continuous infusion) was given since IVIG can inhibit Fas-FasL interaction and halt the progression of SJS. HLH was suspected when the patient’s condition continued to deteriorate with hyperpyrexia and pancytopenia. Routine blood monitoring revealed anemia (hemoglobin 79 g/L), leukopenia (white blood cell count 0.6 × 10^9^/L) and agranulocytosis (neutrophils 0.01 × 10^9^/L). Then the HLH was suspected and the ferritin, NK cells and soluble CD25 were measured. Also the bone marrow smear was performed.

**Figure 2:**
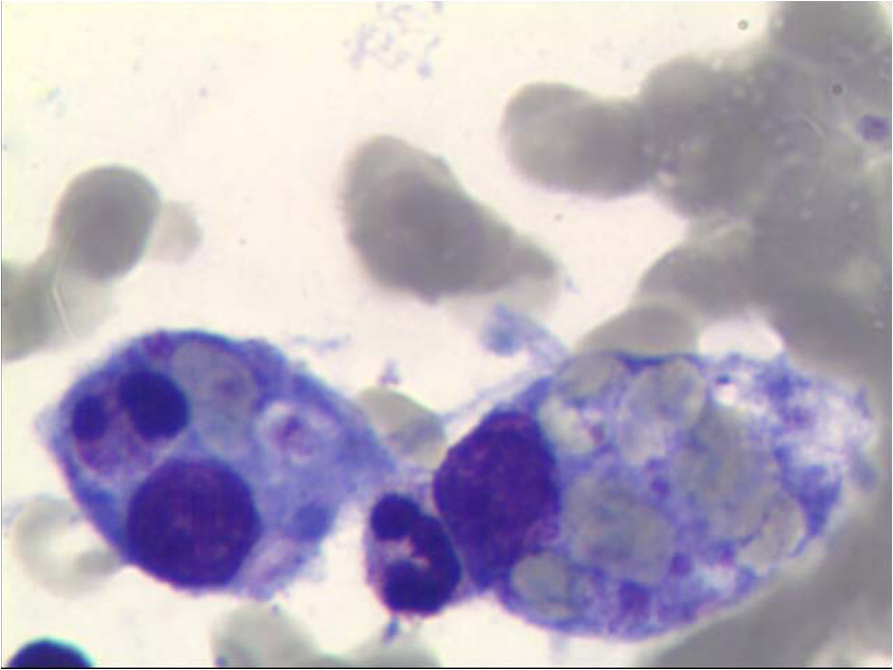
Hemophagocytosis in the bone marrow in our patient.

The diagnosis of SJS-associated HLH was established since the patient fulfilled 6 out of 8 HLH-2004 diagnostic criteria (Table 1) [4]. The laboratory tests showed hyperferritinemia (1031 μg/L), increased soluble CD25 level (8910 U/ml), decreased NK cell activity (1.02%) and hemophagocytosis in bone marrow (Figure 2).

**Table 1:** Clinical and laboratory parameters at diagnosis and after remission according to HLH-2004 guidelines

**Criteria**	**At diagnosis**	**After remission**	**Reference values**
1. Fever	Yes	No	
2. Splenomegaly	No	No	
3. Cytopenias (≥2 lineages)			
Hemoglobin (g/L)	79	125	<90
Platelets (×10^9^/L)	198	332	<100
Neutrophils (×10^9^/L)	0.01	2.38	<1.0
4. Hypertriglyceridemia and/or hypofibrinogenemia			
Triglycerides (mmol/L)	1.98	0.69	≥3.0
Fibrinogen (g/L)	4.2	5.3	≤1.5
5. Hemophagocytosis	Yes (bone marrow)		
6. NK cell activity (%)	1.02	19.39	≤8
7. Ferritin (μg/L)	1031	259	≥500
8. Soluble CD25 (U/ml)	8910	1341	≥2400

Then pulse methylprednisolone (15 mg/kg/day) was administered for 3 consecutive days. The syndrome was considered to be in remission when the patient’s general condition improved, with normal temperature, ameliorated rash and normal routine blood examinations. Hereafter, methylprednisolone was continued at 2.5 mg/kg/day for another 3 days followed with appropriate rapid tapering by 20 mg every 3 days. His condition progressively improved, with normal hematological and biochemical laboratory parameters, inflammatory indices and ferritin level. On the day before discharge in good condition, the bone marrow aspiration was performed showing the absence of hemophagocytosis. Some known genetic defects including PRF1, UNC13D and STX11 were examined to exclude the familial HLH but no mutations were found.

## Discussion

Secondary HLH has been described mainly in association with viral, bacterial, fungal, and parasitic infections, malignant or rheumatologic disorders [4]. There is substantial mortality in secondary HLH, depending on the underlying disease [3]. So far, HLH has seldom been reported in patients with SJS. It should be noted that both SJS and HLH have a high mortality rate, so concurrence of the two in a patient is especially danger. Matsumoto Y. et al. [5] reported a 34-year-old woman who presented SJS complicated with HLH. Although repeated plasmapheresis was conducted, the woman still died of a brain hemorrhage due to disseminated intravascular coagulation.

Until recently, the pathophysiological mechanisms of secondary HLH are incompletely elucidated. The degree of cytokine disturbance is closely related to clinical signs and symptoms [6]. HLH often carries a very bad prognosis for being underdiagnosed and undertreated [7]. At the early stage before proinflammatory damage and cytokine storm, the opportunity to prompt recovery in effective treatment was often missed [8]. Furthermore, it is important for clinicians to differentiate HLH from underlying disease flares or infectious complications because they may share nearly all clinical and laboratory characteristics.

The timely awareness and diagnosis of HLH is the first, crucial step toward successfully life-saving therapy but is challenging because HLH is always complicated by relatively non-specific and variable presentations. NK-cell activity is typically low or absent in HLH, and should be also measured post remission [9]. CD25 is a useful inflammatory marker correlating with disease activity. But obtaining these two results required time. Hyperferritinemia is proved to be a valuable disease marker of HLH. An elegant study by Allen CE et al. [10] showed that a ferritin level over 10,000 ng/ml in children was 90% sensitive and 96% specific for HLH. Hemophagocytosis is important in the diagnostic work-up of HLH [4]. However, the sensitivity of hemophagocytosis was 83% with a specificity of only 60% for HLH [11]. Moreover, hemophagocytosis may be lacking early at onset. Waiting for evidence of hemophagocytosis to make therapeutic decisions in the bone marrow may delay appropriate therapy in this life-threatening condition [12]. It is important to note that these diagnostic criteria are mainly helpful in making the diagnosis instead of reflecting all of the typical clinical or laboratory features of patients with HLH. Of the ubiquitous responses in HLH, the pancytopenia is the most common feature and this can be easily detected by routine blood monitoring. HLH diagnosis is based on progressive cytopenia affecting at least two cell lineages. Considering the incidence of pancytopenia is very low in SJS, it can be an early indicator for SJS complicated with HLH and enables clinicians to start a prompt diagnosis and a timely life-saving therapy.

Therapeutic strategies for secondary HLH are not well established because randomized controlled clinical trails are not available [13]. However, based on our own experience [14] and some multi-center cohort studies [15], the initial treatment should be capable of suppressing the severe hyper-inflammation and controlling the underlying disease. A recent analysis of our experience treating 23 secondary HLH patients (so-called macrophage activation syndrome) triggered by systemic onset juvenile idiopathic arthritis, Kawasaki disease and systemic lupus erythematosus showed that 21 children were successfully treated with IVIG, corticosteroid and cyclosporine A. Corticosteroid and/or immunosuppressants seem to be the mainstay of treatment for secondary HLH [16]. When the trigger event is SJS per se in our patient, IVIG and corticosteroid are proposed to be first line therapeutic tools according to the HLH-2004 protocol [4]. High-dose IVIG can be given before a definite diagnosis of HLH since IVIG can halt the progression of SJS. In addition, we deemed that early use of IVIG may prevent a cytokine storm in HLH through the blockade of Fc receptors and other pathways. After establishing diagnosis of secondary HLH, the corticosteroid should be used to counteract the activated macrophages and stop the hyperinflammation stage of the syndrome.

## Conclusions

In conclusion, HLH in SJS is very uncommon and the condition is life-threatening. IVIG in combination with corticosteroid is a safe and effective therapeutic regimen. Keeping highly vigilant to make an early diagnosis is essential to ensure favorable outcomes. Pancytopenia can be a precocious indicator and enables to start a prompt diagnosis and treatment.

## Consent

Written informed consent was obtained from the patient’s parents for publication of this Case report and any accompanying images. A copy of the written consent is available for review by the Editor-in-Chief of this journal.

## Abbreviations

SJS: Stevens-Johnson syndrome; HLH: Hemophagocytic lymphohistiocytosis; IVIG: Intravenous immunoglobulin; SHLH: Secondary hemophagocytic lymphohistiocytosis.

## Competing interests

The authors declare that they have no competing interests.

## Authors’ contributions

ZDF and XQQ drafted the manuscript. HGY made a critical revision. All authors read and approved the final manuscript.

## Pre-publication history

The pre-publication history for this paper can be accessed here:

http://www.biomedcentral.com/1471-2431/14/38/prepub
